# Supplementary material for: Identification of Vulnerable Cell Types in Major Brain Disorders Using Single Cell Transcriptomes and Expression Weighted Cell Type Enrichment
Source: Front Neurosci. 2016 Jan 27;10:16. doi: 10.3389/fnins.2016.00016 (PMC4730103; doi:10.3389/fnins.2016.00016)
Supplement: Supplementary file 3 [file SupplementaryMethods.PDF]

**Sources of Transcriptome Datasets**

Alzheimer's:

Publication: PMID17845826[1]

Source: Author maintains on a private server. Details provided by email.

Brain regions: *frontal pole, occipital cortex primary visual cortex, inferior temporal gyrus, middle temporal gyrus, superior temporal gyrus, posterior cingulate cortex, anterior cingulate, entorhinal cortex, temporal pole, precentral gyrus, inferior frontal gyrus. dorsolateral refrontal cortex, superior parietal lobule, superior frontal gyrus, caudate nucleus, hippocampus, putamen*

Technology platform: Affymetrix Human Genome U133A + U133B Arrays

Analysis notes:

Raw data was read into R from .cel files using the affy package[2] and normalized using the RMA package. Probe annotations were obtained through Biomart. Linear models were fitted to the expression values for each probe using limma[3], with Braak stage as the independent variables and sex, age, and ancestry taken as extraneous variables. The U133A and U133B arrays were analysed separately and then merged by combining the rows from both studies into one table.

Autism:

Publication: PMID25494366[4]

Source: http://www.arkinglab.org/resources/

Brain regions: associative visual cortex, frontal pole, inferior frontal gyrus

Technology platform: RNA-Seq

Analysis notes:

The preprocessed data was downloaded from the lab webpage as 'Samples104BGenes-EDASeqFull.txt'. A linear model was fitted to the expression values for each probe using limma[3], with disease status as the independent variables and brain region, age, and processing site and sex taken as extraneous variables. Age was treated within the model as a spline with three degrees of freedom.

Publication: PMID21614001[5]

Source: GSE28521

Brain regions: superior temporal gyrus, dorsolateral prefrontal cortex

Technology platform: Illumina HumanRef-8 v3.0

Analysis notes:

Normalised data was obtained from GEO. Probe annotations were obtained through Biomart. A linear model was fitted to the expression values for each probe using limma[3], with disease status as the independent variables and brain region as an extraneous variable.

Schizophrenia:

Publication: PMID25796564[6]

Source: GSE53987

Brain regions: prefrontal cortex, hippocampus, striatum

Technology platform: Affymetrix Human Genome U133 Plus 2.0

Analysis notes:

Raw data was read into R from .cel files using the affy package[2] and normalized using the RMA package. Probe annotations were obtained through Biomart. Linear models were fitted to the expression values for each probe using limma[3], with disease status as the independent variables with sex and age as extraneous variables. Schizophrenia and Bipolar Disorder samples were both studied and the later treated as schizophrenia samples in the later analysis.

Publication: PMID16139990[7]
Source: Author maintains on a private server. Details provided by email.
Brain regions: *frontal pole, occipital cortex primary visual cortex, inferior temporal gyrus, middle temporal gyrus, superior temporal gyrus, posterior cingulate cortex, anterior cingulate, entorhinal cortex, inferior frontal gyrus, dorsolateral prefrontal cortex, superior parietal lobule, superior frontal gyrus, caudate nucleus, hippocampus, putamen*
Technology platform: Affymetrix Human Genome U133A + U133B Arrays
Analysis notes:

Raw data was read into R from .cel files using the affy package[2] and normalized using the RMA package. Probe annotations were obtained through Biomart. Linear models were fitted to the expression values for each probe using limma[3], with disease status as the independent variables and sex and age taken as extraneous variables. The U133A and U133B arrays were analysed separately and then merged by combining the rows from both studies into one table.

Publication: PMID22868662[8]
Source: Author maintains on a private server. Details provided by email.
Brain regions: *middle temporal gyrus, anterior cingulate, temporal pole, dorsolateral prefrontal cortex*
Technology platform: Affymetrix Human Genome U133 Plus 2.0
Analysis notes: Raw data was read into R from .cel files using the affy package[2] and normalized using the RMA package. Probe annotations were obtained through Biomart. Linear models were fitted to the expression values for each probe using limma[3], with disease status as the independent variables with sex and age as extraneous variables.

Publication: PMID21538462[9]
Source: GSE21935
Brain regions: *superior temporal gyrus*
Technology platform: Affymetrix Human Genome U133 Plus 2.0
Analysis notes: Raw data was read into R from .cel files using the affy package[2] and normalized using the RMA package. Probe annotations were obtained through Biomart. Linear models were fitted to the expression values for each probe using limma[3], with disease status as the independent variables with sex and age as extraneous variables.

Publication: PMC2783475[10]
Source: GEO21138

Brain regions: *dorsolateral prefrontal cortex*
Technology platform: Affymetrix Human Genome U133 Plus 2.0 Array
Analysis notes: Processed data was downloaded from GEO. Probe annotations were obtained through Biomart. Linear models were fitted to the expression values for each probe using limma[3], with disease status as the independent variables. Actual ages were not provided with the dataset, however three catagories of how long the disease had been diagnosed for were provided. These three catagories were used as a proxy for age as an extraneous variable.

Publication: PMID19255580[11]
Source: GEO17612
Brain regions: *frontal pole*
Technology platform:
Analysis notes: Raw data was read into R from .cel files using the affy package[2] and normalized using the RMA package. Probe annotations were obtained through Biomart. Linear models were fitted to the expression values for each probe using limma[3], with disease status as the independent variables with sex and age as extraneous variables.

## References


[1] V. Haroutunian, P. Katsel, and J. Schmeidler, Transcriptional vulnerability of brain regions in Alzheimer's disease and dementia. Neurobiology of aging 30 (2009) 561-573.

[2] L. Gautier, L. Cope, B.M. Bolstad, and R.A. Irizarry, affy—analysis of Affymetrix GeneChip data at the probe level. Bioinformatics 20 (2004) 307-315.

[3] M.E. Ritchie, B. Phipson, D. Wu, Y. Hu, C.W. Law, W. Shi, and G.K. Smyth, limma powers differential expression analyses for RNA-sequencing and microarray studies. Nucleic acids research (2015) gkv007.

[4] S. Gupta, S.E. Ellis, F.N. Ashar, A. Moes, J.S. Bader, J. Zhan, A.B. West, and D.E. Arking, Transcriptome analysis reveals dysregulation of innate immune response genes and neuronal activity-dependent genes in autism. Nature communications 5 (2014).

[5] I. Voineagu, X. Wang, P. Johnston, J.K. Lowe, Y. Tian, S. Horvath, J. Mill, R.M. Cantor, B.J. Blencowe, and D.H. Geschwind, Transcriptomic analysis of autistic brain reveals convergent molecular pathology. Nature 474 (2011) 380-384.

[6] V. Reinhart, S.E. Bove, D. Volfson, D.A. Lewis, R.J. Kleiman, and T.A. Lanz, Evaluation of TrkB and BDNF transcripts in prefrontal cortex, hippocampus, and striatum from subjects with schizophrenia, bipolar disorder, and major depressive disorder. Neurobiology of disease 77 (2015) 220-227.

[7] P. Katsel, K.L. Davis, and V. Haroutunian, Variations in myelin and oligodendrocyte-related gene expression across multiple brain regions in schizophrenia: a gene ontology study. Schizophrenia research 79 (2005) 157-173.

[8] P. Roussos, P. Katsel, K.L. Davis, L.J. Siever, and V. Haroutunian, A system-level transcriptomic analysis of schizophrenia using postmortem brain tissue samples. Archives of general psychiatry 69 (2012) 1205-1213.
[9] M.R. Barnes, J. Huxley - Jones, P.R. Maycox, M. Lennon, A. Thornber, F. Kelly, S. Bates, A. Taylor, J. Reid, and N. Jones, Transcription and pathway analysis of the superior temporal cortex and anterior prefrontal cortex in schizophrenia. Journal of neuroscience research 89 (2011) 1218-1227.
[10] S. Narayan, B. Tang, S.R. Head, T.J. Gilmartin, J.G. Sutcliffe, B. Dean, and E.A. Thomas, Molecular profiles of schizophrenia in the CNS at different stages of illness. Brain research 1239 (2008) 235-248.
[11] P.R. Maycox, F. Kelly, A. Taylor, S. Bates, J. Reid, R. Logendra, M.R. Barnes, C. Larminie, N. Jones, and M. Lennon, Analysis of gene expression in two large schizophrenia cohorts identifies multiple changes associated with nerve terminal function. Molecular psychiatry 14 (2009) 1083-1094.
